# Antimicrobial Susceptibility of *Salmonella* Isolated from Chickens and Humans in Wau, South Sudan

**DOI:** 10.1155/2022/8570081

**Published:** 2022-11-30

**Authors:** Shereen Saad, Ambros Jubara, Charles Wani, Musso Munyeme, Alfateh Taha, Steward Mudenda, Mildred Zulu, Wizaso Mwasinga, Mulemba Samutela, Rabecca Tembo, Walter Muleya, Geoffrey Kwenda, Bernard Mudenda Hang'ombe

**Affiliations:** ^1^Department of Clinical Studies, University of Bahr El Ghazal, College of Veterinary Science, Wau, Sudan; ^2^Department of Disease Control, School of Veterinary Medicine, University of Zambia, Lusaka, P.O. Box 32379, Zambia; ^3^Department of Pharmacy, School of Health Sciences, University of Zambia, Lusaka, P.O. Box 50110, Zambia; ^4^Department of Pathology and Microbiology, School of Medicine, University of Zambia, Lusaka, P.O. Box 50110, Zambia; ^5^Department of Biomedical Sciences, School of Health Sciences, University of Zambia, Lusaka, Zambia; ^6^Department of Biomedical, School of Veterinary Medicine, University of Zambia, Lusaka, P.O. Box 32379, Zambia; ^7^Department of Paraclinical Studies, School of Veterinary Medicine University of Zambia, Lusaka, Zambia

## Abstract

**Background:**

*Salmonella* infections are a public health problem across the globe. In South Sudan, there is little information regarding the prevalence and antibiotic resistance patterns of *Salmonella*. Therefore, this study assessed the prevalence and antimicrobial susceptibility of *Salmonella* isolates from chickens and humans in South Sudan. Fecal samples were collected and cultured on Xylose Lysine Deoxycholate Agar for the isolation of *Salmonella* and confirmed using biochemical tests and PCR through the amplification of the *invA* gene. A total of 417 fecal samples were examined, of which 270 (64.7%) were chicken cloacal swabs while 147 (35.3%) were humans' stool specimens.

**Results:**

Eleven (11) *Salmonella* isolates were isolated from humans while nine were from chickens. All 11 isolates from humans were susceptible to sulfamethoxazole-trimethoprim, chloramphenicol, streptomycin, cefotaxime, nalidixic acid, and gentamicin. However, 4 (36.7%) isolates showed resistance to ciprofloxacin, 2 (18.9%) to ampicillin, and 1 (9.1%) to tetracycline. All chicken isolates were susceptible to chloramphenicol, streptomycin, sulfamethoxazole-trimethoprim, ciprofloxacin, cefotaxime, nalidixic acid, and gentamicin but showed resistance to tetracycline 2 (22.2%) and ampicillin 1 (11.1%).

**Conclusion:**

Antimicrobial resistant isolates were isolated in both chickens and humans. Further, MDR isolates were found in both chicken and human samples, and this is a public health concern. This, therefore, calls for concerted efforts to educate producers and consumers on public health, food safety, food hygiene in food production, and enhancement of surveillance programmes on zoonotic bacteria and antimicrobial susceptibility.

## 1. Introduction

Worldwide, *Salmonella* has been listed among the most important food-borne pathogen that is transmitted through the consumption of contaminated food [1]. It causes approximately 1.4 million cases of disease and about 20,000 hospital cases and over 500 deaths every year [2]. A growing number of human Salmonellosis cases have been associated with the consumption of contaminated food of poultry origin, such as chicken and chicken products [3]. Besides, chicken products have also been reported to play a major role in the spreading of antimicrobial-resistant zoonotic bacterial pathogens [4] even though the hygienic standards for chicken production are quite high and usually vary from place to place [5, 6]. The problem of AMR is still rising.

Antimicrobial resistance (AMR) and particularly multidrug resistance (MDR) is becoming very common among various *Salmonella* serotypes that have been isolated from humans and chickens world over [7]. The extent of AMR varies from region to region and is usually influenced by the abuse of antibiotics in humans and animals [8]. Reports of cases of *Salmonella* isolates being resistant to important antibiotics have been reported dating back to the 1960s during which resistance was reported to have been limited to one antibiotic [9]. However, from the 1970s onwards, there has been an increase in the number of *Salmonella* isolates that have shown resistance to various clinically significant antibiotics, and this has been exacerbated by the recovery of such isolates in foods of animal origin [10]. This is a growing public health concern as human Salmonellosis caused by resistant strains of *Salmonella* may be difficult to be treated [10]. Since the mid-1970s, there has been an increasing trend of *Salmonella* isolates exhibiting MDR phenotypes worldwide [11]. The MDR exhibited by some *Salmonella* isolates and other pathogens is obtained from extrachromosomal genes that may impart resistance to an entire class of antimicrobials [12]. More recently, most of the resistance genes have been associated with large transferable plasmids and other DNA mobile elements, such as transposons and integrons [9, 13]. Moreover, MDR seems to be more serious in some serotypes compared to other serotypes [14, 15]. Therefore, there is a need for continuous monitoring of human and animal *Salmonella* isolates that exhibit resistance to most antimicrobials on a global scale [16].

In South Sudan, a young country where everything is still in its infancy, there is limited information regarding *Salmonella* species which was confirmed by PCR amplification of the invA gene. Therefore, this study assessed the prevalence and antimicrobial susceptibility of *Salmonella* isolated from humans and chickens in Wau, Western Bahr el Ghazal state, South Sudan, to inform control strategies.

## 2. Results

### 2.1. Isolation of *Salmonella* from Fecal Samples

The overall prevalence of *Salmonella* in the study was 4.8% (20/417), as shown in Table 1. The prevalence was relatively higher in humans, about 7.5% (11/147) compared to 3.3% (9/270) in chickens (Table 1). The *Salmonella* was further confirmed by PCR using the *invA* gene.

The prevalence of *Salmonella* in humans and chickens based on areas where samples were collected is shown in Table 2. The highest prevalence among human samples was from Baggari (9.4%) followed by Busuri (4.8%) (Table 2). For chicken samples, Baggari showed the highest prevalence of 4.5.% while Busuri payam showed a relatively lower prevalence of 1.7% (Table 2).

### 2.2. Detection of Extended Spectrum Beta-Lactamases (ESBLs) in *Salmonella* Strain

All the 20 *Salmonella* isolated from chickens and chicken keepers (humans) did not show any sign of growth on MacConkey agar supplemented with 2 mg/l of cefotaxime implying that all the 20 isolates were susceptible to cefotaxime.

### 2.3. *Salmonella* Serotypes Isolated from Human and Chickens

The *Salmonella* isolated from humans and chicken belonged to five serotypes, namely, *Salmonella* Aberdeen, Enteritidis, Uganda, Montevideo, and Typhimurium (Table 3). *Salmonella* Typhimurium was the most detected serotype (Table 3). Some nontypeable isolates were detected in humans while *Salmonella* Uganda and *Salmonella* serovar Montevideo were found in chickens only (Table 3).

### 2.4. Antimicrobial Susceptibility Patterns of *Salmonella* Isolates from Humans and Chickens

**Figure 1:**
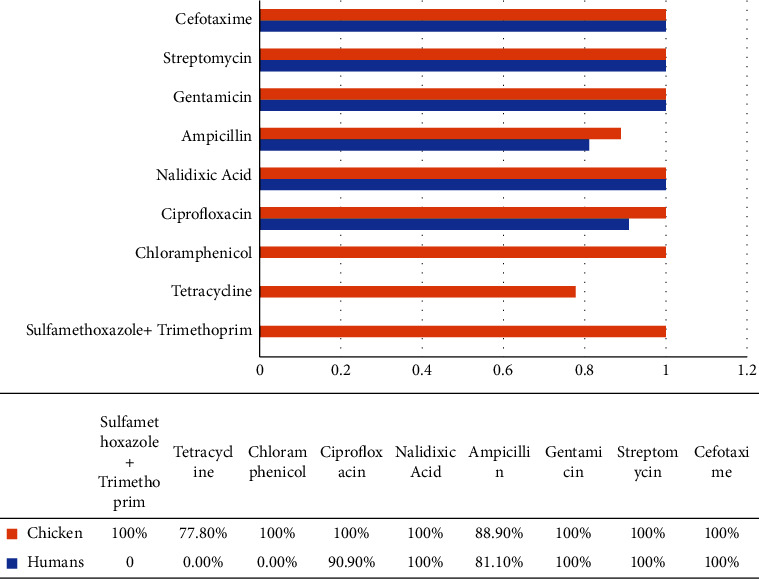
Antimicrobial susceptibility profiles of *Salmonella* isolates from humans and chickens.

**Figure 2:**
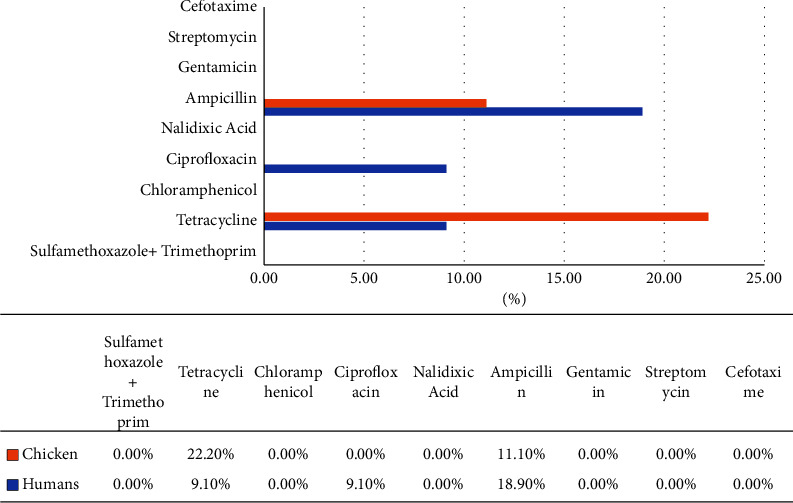
Continued Antimicrobial resistance profiles of *Salmonella* isolates from humans and chickens.

Of the 11 isolates obtained from the 147 human samples, seven isolates showed 100% susceptibility to the following drugs sulfamethoxazole/trimethoprim, chloramphenicol, streptomycin, nalidixic acid, cefotaxime, and gentamicin (Figure 1). However, some isolates showed resistance to ciprofloxacin 1 (9.1%), tetracycline 1 (9.1%), and ampicillin 2 (18.2%) (Figure 2). All the chicken isolates (100%) were susceptible to chloramphenicol, cefotaxime, streptomycin, sulfamethoxazole/trimethoprim, nalidixic acid, and gentamicin (Figure 1). The isolates were resistant to ampicillin (11.1%) and tetracycline (22.2%) (Figure 2).

### 2.5. Antimicrobial Resistance Profiles of *Salmonella* Isolates

Antimicrobial resistance profiles of the 20 *Salmonella* isolates were divided into three different MDR resistotype profiles (Table 4). This resistance was based on resistance to at least three different antimicrobials [17]. Resistotype group 1, resistance to ampicillin, tetracycline, ciprofloxacin, and gentamicin, was represented by 3 (15%) isolates. Resistotype group 2, resistance to ampicillin, gentamicin, and tetracycline, was detected in only 1 (5%) isolates, specifically *Salmonella* Typhimurium isolated from humans and chickens. However, resistotype group 3, resistance to ampicillin and ciprofloxacin, was found in three (15%) isolates. *Salmonella* strains isolated from the chicken source were represented in two groups of the three MDR resistotypes 1 and 2. The *S*. Enteritidis strain from the humans and chickens was resistant to two antimicrobials, namely, ampicillin and tetracycline.

## 3. Discussion

The present study investigated *Salmonella* in chickens and humans in Wau town of South Sudan. A previous study focused on *Salmonella* from chickens with prevalence of 1.1% [18]. Recently in 2017, Shereen Saad and collegues (unpublished work) investigated the prevalence of *Salmonella* and antimicrobial resistance in humans and chickens. However, the previous study did not involve molecular confirmation; as such, the study could not identify the serotypes circulating in the study area. The present study thus utilized molecular techniques to identify the different serotypes of *Salmonella* circulating present in both chicken keepers and chickens reared in Baggari and Busari regions of South Sudan.

*Salmonella* is an important cause of morbidity and mortality in human and animals and has thus emerged as a significant and growing public health and economic problem worldwide [19]. The overall prevalence of *Salmonella* in this study was 7.5% and 3.3% for humans and chicken, respectively. Human isolates showed its higher prevalence compared to chicken isolates. This difference may have resulted from the bigger volume of human sample (stool) in comparison to the chicken droopings. This was lower when compared to a similar study conducted in Khartoum, Sudan, where the prevalence was 70.1% in chicken handlers and 18.1% in chickens [20]. The difference in the prevalence was found in the current study and the previous study might be due to a larger sample size (996) that involved pooling of samples in the previous study. However, the higher prevalence of 68.2%, 72%, 25.9%, and 51.2% have been reported in Ethiopia, Thailand, Korea, and Argentina, respectively [21].

*Salmonella* isolated from chickens and chickens keepers were screened to determine AMR patterns. Three (3, 27.2%) isolates from chicken keepers were resistant to more than one antibiotic. This was not in agreement with the findings obtained by Fadlalla et al., where the resistance of *Salmonella* was observed in 81 human samples (93.1%) [22]. The present study further indicated that 1 (9.1%) of the human *Salmonella* isolates was highly resistant (>0.3) to tetracycline and ciprofloxacin while 2 (18.9%) were resistant to ampicillin. However, another study conducted in Sudan reported higher resistance patterns, including resistance to ampicillin 29 (33.3%), nalidixic acid 28 (32.2%), and tetracycline 52 (59.8%) compared to the ones reported in the present study [22]. Furthermore, another study conducted in Sudan also reported higher resistance profiles of *Salmonella* isolates to tetracycline 11.8% and sulfamethoxazole/trimethoprim 88.2% [20].

The current study has also indicated that 7 (63.6%) isolates were 100% susceptible to sulfamethoxazole/trimethoprim, chloramphenicol, cefotaxime, streptomycin, nalidixic acid, and gentamicin. This was in agreement with the results of Fadalall et al.'s study [22] except for some isolates that exhibited moderate resistance (>0.22) to ciprofloxacin 1 (9.1%). Furthermore, the results of the current study are also congruent with a study conducted in Brazil, where 88.2% of *Salmonella* isolates showed resistance to sulphonamides [23]. This resistance of *Salmonella* isolates to most of the antimicrobials could be due to the presence of resistance genes that are carried on the bacterial plasmids which can be acquired by the consumption of contaminated animal products by humans [24]. Continuous use of antibiotics such as ampicillin and nalidixic acid as treatment remedies may also be the reason for development of resistance [5]. Chloramphenicol combined with ampicillin has been widely used for the treatment of human salmonellosis despite this combination being known for causing aplastic anaemia. For example, it has been a drug of choice in Brazil since 1970s [23].

Salmonellosis in humans has been a health problem in both developed and undeveloped countries with nontyphoidal *Salmonella* (NTS) caused by other species of *Salmonella* different from *S. typhi* being a major cause of secondary bacteremia associated with gastritis [19]. In the present study, *S. Enteritidis* and *S. Typhimurium* were commonly isolated in chicken keepers while nontypeable isolates of *Salmonella* were the most dominant serovars. Generally, these serovars showed low resistance to tetracycline and high resistance to ciprofloxacin and ampicillin which are the antibiotics commonly used in South Sudan.

## 4. Conclusions

Antimicrobial resistant isolates were isolated in both chickens and humans. Further, MDR isolates were found in both chicken and human samples which is a public health concern. This, therefore, calls for concerted efforts to educate consumers on public health, food safety, food hygiene in food production, and enhancement of surveillance programmes on zoonotic bacteria and antimicrobial susceptibility.

## 5. Materials and Methods

### 5.1. Study Site

**Figure 3:**
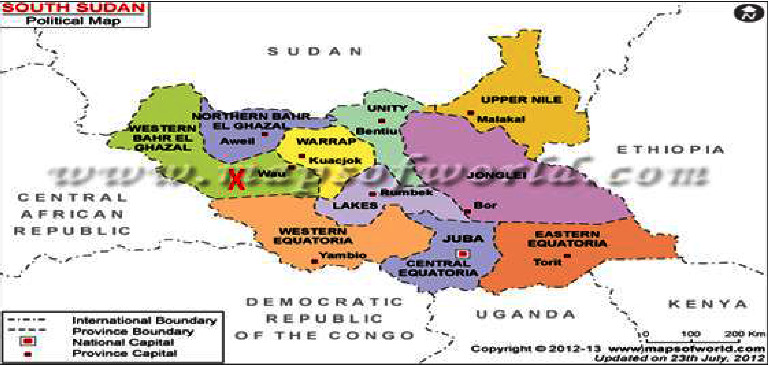
Study area highlighted by X (Google Map).

The study was conducted in the Western Bahr El Ghazal State, which shares boundaries with Sudan to the North and Central African Republic to the West with coordinates of 8.6452°N, 25.2838°E, and 626.9 meters above the sea level (Figure 3, study area map). The climate is tropical with an annual rainfall ranging between 400 and 1600 mm and temperature of 23.8°C–40°C. Of the five payams of Wau County, two payams, namely, Baggari and Bussuri were randomly selected for the study.

### 5.2. Study Design

The study design employed was a cross-sectional survey design targeting local live chickens and humans at the household level in Wau County, Western Bahr el Ghazal State, South Sudan. Sample collection was carried out between September and December 2019.

### 5.3. Sample Size and Sampling Technique

Using simple random sampling, a total of 270 cloacal swabs and 147 fecal samples were collected from local live chickens and humans, respectively. The samples were collected with sterile wooden swabs from the chicken cloacal, transferred into 10 ml sterile universal containers containing the 5 ml Cary Blair Transport Medium (Himedia), and immediately placed into a cool box containing ice packs. The samples were then transported and stored in a refrigerator at 4°C till analysis.

Before collecting human stool samples, consent was sought from community gatekeepers such as chiefs, opinion leaders, and elders. Additional consent was collected from human participants as a requirement for ideal sample collection. Stool samples (147) were carefully collected into sterile 10 ml plastic universal containers with spoons to which 5 ml Cary Blair Transport Medium has been added. The samples were then transported and stored in a refrigerator at 4°C until analysis.

### 5.4. Isolations and Identifications of *Salmonella* Species from the Fecal Sample

The samples were inoculated and incubated at 37°C in preenriched, nonselective buffered peptone water for 24 hours. An aliquot from peptone water (1 ml) was cultured in Rappaport-Vassiliadis broth in the ration of 1 to 10 for each. From the broth, a loopful was innoculated on Xylose Dextrose Agar (XLD, Oxoid, UK), The temperature and the period of incubation was done at 37°C for 24 hours for both chicken droppings and human stool samples [25, 26]. The suspected colonies of *Salmonella* from each plate were collected for presumptive identification by biochemical tests that included oxidase, hydrogen sulphide (H_2_S), urease, indole, and fermentation of glucose, sucrose, mannitol, and lactose [27]. Furthermore, S*almonella* was confirmed using PCR targeting the *invA*, as previously described [28].

### 5.5. Serological Typing of *Salmonella* Isolates

The *Salmonella* isolates were characterized into sero groups based on the presence of distinctive “O” antigenic factor, using O polyvalent antiserum and specific monovalent antiserum for A-S group antigen and GROUP BC1, C2, D1, E, and G, respectively, in accordance to the manufacture's protocol for the identification of surface antigens and their differentiation into serogroups. Briefly, a drop of the appropriate antiserum was placed onto a clean microscopic slide. A single colony of overnight culture on nutrient agar was picked and emulsified in the antiserum drop to obtain a thoroughly mixed suspension. The slide was gently rocked forward and backwards/side wards for 1 minute. Agglutination or clumping between 1 and 10 seconds was considered as a positive reaction.

### 5.6. Serotyping of *Salmonella* Strains

*Salmonella* strains were differentiated into serotypes by serotyping analysis according to the method described by [28, 29]. All isolates of *Salmonella* was referred to Deltamune (Pty) Laboratory, a South African SANAS Accredited Veterinary Laboratory, for confirmation and serotyping. Characterization was done using the method described in the Microbiological Manual, and serotyping (10.2 : 1995 CCFRA) was done based on White-Kauffman-Le Minor Scheme (WHO Collaborating Center) [30]. The scheme discriminated serotypes on the basis of their somatic (O), flagella (H), and capsular (Vi) antigens present on the surface of *Salmonella* [31].

### 5.7. DNA Extraction for *Salmonella* Confirmation

The bacteria were cultured on nutrient agar for 24 hrs at 37°C and DNA extraction was performed by the boiling method for 10 min and centrifugation at 5000 ×g for 5 min. The supernatant was then used for the DNA amplification using *Salmonella*-invA gene specific primers, namely, S139 (5′GTG AAA TTA TCG CCA CGT TCG GGC AA -3′ and S141 (5′ TCA TCG CAC CGT CAAAGG AAC C -3′), as described previously [32]. The reaction volume was 25 *μ*l with 1 *μ*l of the DNA template. The following PCR conditions were used: 94°C for 60 sec of the initial denaturation followed by 30 cycles of 60 sec at 94°C, 30 sec at 56°C, 30 sec at 72°C, 2 min and a final extension step of 10 min at 72°C. The amplified PCR products were then visualised on 1.5% agarose gel stained with ethidium bromide and visualised by UV illumination alongside a 100 bp DNA ladder.

### 5.8. Detection of Extended Spectrum Beta-Lactamases (ESBLs) in the *Salmonella* Strain

Detection of extended spectrum cephalosprinase production isolate was accomplished using freshly prepared MacConkey Agar (HIMEDIA) containing 2 mg/l of cefotaxime (Sigma-Aldrich, Munich, Germany) according to the method described in [28].

### 5.9. Determination of Antimicrobial Susceptibility Patterns of *Salmonella* Isolates

The antibiotic susceptibility profiling of the *Salmonella* isolates was determined using the Kirby–Bauer disc diffusion method based on the Clinical Laboratory Standard Institute (CLSI) guidelines [33]. The antibiotic discs (Oxoid, UK) included ampicillin, sulfamethoxazole/trimethoprim, streptomycin, ciprofloxacin, cefotaxime, tetracycline, gentamicin, nalidixic acid, and chloramphenicol. Using CLSI guidelines that provide ranges for zone of inhibition, the AST on all isolates were read and grouped into Susceptible (S), Intermediate (I), and Resistance (R), and for quality control purpose, *E. coli* ATCC 25922 was used.

### 5.10. Determination of Multiple Antimicrobial Resistances Indexing MARI

The multiple antibiotic resistance index was calculated as follows: *a*/*b*, where “*a*” represents the number of antibiotics to which the particular isolate was resistant and “*b*” the number of antibiotics to which the isolate was exposed. MARI values >0.2 are considered significant indicating that the strains could have originated from sources where antibiotics are often used [34]. While MARI value <0.2 suggests the strains originate from animal sources which are less frequent exposed to antibiotics or never at all [35].

### 5.11. Data Analysis

Data analysis was done using Statistical Package for Social Sciences (SPSS) version 22.

## Figures and Tables

**Table 1:** Isolation of *Salmonella* from fecal samples.

Host	Total tested *n* (%)	Positive *n* (%)	Negative *n* (%)
Human	147 (35.3)	11 (7.5)	136 (92.5%)
Chicken	270 (64.7)	9 (3.3)	291 (96.7%)

**Table 2:** Prevalence of *Salmonella* in humans and chicken per sampling area.

Sampling area	Humans (*n*/total)	Prevalence among humans (%)	Chickens (*n*/total)	Prevalence among chickens (%)
Baggari	85	8 (9.4%)	157	7 (4.5%)
Busuri	62	3 (4.8%)	113	2 (1.7%)

**Table 3:** *Salmonella* serotype isolated from humans and chickens.

S/N	Isolate	Humans host	Chicken host	Total
1	*Salmonella* Aberdeen	3	1	4
2	*Salmonella* Enteritidis	1	2	3
3	*Salmonella* Uganda		1	1
4	Nontypeable	5		5
5	*Salmonella* Typhimurium	2	4	6
6	*Salmonella* serovar Montevideo		1	1
Total		11	09	20

**Table 4:** Antimicrobial resistance profiles of *Salmonella* isolates.

No. of strains	Serotype	Resistotype	Group No	Host
3	Enteritidis	Amp, Te, Cip, Gn	1	Humans/chickens
1	*Salmonella* Typhimurium	Cip, Gen, Te	11	Humans/chickens
3	Nontypeable	Amp, Cip	111	Humans

## Data Availability

All data used for the study are available upon request from the corresponding author.

## Supplementary Materials

Supplementary Materials Supplementary material 1: the table shows the sample culture results as obtained from the XLD media. Supplementary material 2: the table shows results of subjecting *Salmonella* isolates to different sugars (Biotyping). Supplementary material 3: the table shows the result obtained from PCR method targeting the *Salmonella InvA* gene. Supplementary material 4: the table provides the inhibition zones and their interpretation results (Antimicrobial Susceptibility Test results).
