# Supplementary material for: Antimicrobial Susceptibility of Salmonella Isolated from Chickens and Humans in Wau, South Sudan
Source: Int J Microbiol. 2022 Nov 30;2022:8570081. doi: 10.1155/2022/8570081 (PMC10287519; doi:10.1155/2022/8570081)
Supplement: Supplementary Materials — Supplementary material 1: the table shows the sample culture results as obtained from the XLD media. Supplementary material 2: the table shows results of subjecting Salmonella isolates to different sugars (Biotyping). Supplementary material 3: the table shows the result obtained from PCR method targeting the Salmonella InvA gene. Supplementary material 4: the table provides the inhibition zones and their interpretation results (Antimicrobial Susceptibility Test results). [file 8570081.f1.docx]

**Supplementary Materials**

**Supplementary Materials 1: Sample culture results as obtained from the XLD media**

| **S/N** | **Sample ID** | **SOURCE** | **PLACE** | **Colony Morphology** |
| --- | --- | --- | --- | --- |
|  | 14 | Baggari | Human | + |
|  | 27 | Baggari | Human | + |
|  | 32 | Baggari | Human | + |
|  | 37 | Baggari | Human | + |
|  | 48 | Baggari | Human | + |
|  | 61 | Baggari | Human | + |
|  | 66 | Baggari | Human | + |
|  | 70 | Baggari | Human | + |
|  | 81 | Busuri | Human | + |
|  | 95 | Busuri | Human | + |
|  | 96 | Busuri | Human | + |
|  | 149 | Baggari | Chicken | + |
|  | 157 | Baggari | Chicken | + |
|  | 167 | Baggari | Chicken | + |
|  | 179 | Baggari | Chicken | + |
|  | 186 | Baggari | Chicken | + |
|  | 357 | Baggari | Chicken | + |
|  | 358 | Baggari | Chicken | + |
|  | 380 | Busuri | Chicken | + |
|  | 390 | Busuri | Chicken | + |

**Supplementary Materials 2: Biotyping of the isolated *Salmonella***

| **S/N** | **Place** | **SOURCE** | **Colony Morphology** | **PCR INVA Gen** | **Xylose** | **Lactose Monohydrate** | **Mannitol** | **Trehalose** | **Sorbitol** | **Arabinose** | **Maltose Monohydrate** |
| --- | --- | --- | --- | --- | --- | --- | --- | --- | --- | --- | --- |
|  | Baggari | Chicken | +Ve | +Ve | +Ve | +Ve | +Ve | +Ve | +Ve | +Ve | +Ve |
|  | Baggari | Chicken | +Ve | +Ve | +Ve | +Ve | +Ve | +Ve | +Ve | +Ve | +Ve |
|  | Baggari | Chicken | +Ve | __Ve | +Ve | +Ve | +Ve | +Ve | +Ve | +Ve | +Ve |
|  | Baggari | Chicken | +Ve | +Ve | +Ve | +Ve | +Ve | +Ve | +Ve | +Ve | +Ve |
|  | Baggari | Chicken | +Ve | __Ve | +Ve | +Ve | +Ve | +Ve | +Ve | +Ve | +Ve |
|  | Baggari | Chicken | +Ve | +Ve | +Ve | +Ve | +Ve | +Ve | +Ve | +Ve | +Ve |
|  | Baggari | Chicken | +Ve | __Ve | +Ve | +Ve | +Ve | +Ve | +Ve | +Ve | +Ve |
|  | Baggari | Chicken | +Ve | __Ve | +Ve | +Ve | +Ve | +Ve | +Ve | +Ve | +Ve |
|  | Busuri | Chicken | +Ve | __Ve | +Ve | +Ve | +Ve | +Ve | +Ve | +Ve | +Ve |
|  | Busuri | Chicken | +Ve | __Ve | +Ve | +Ve | +Ve | +Ve | +Ve | +Ve | +Ve |
|  | Busuri | Chicken | +Ve | +Ve | +Ve | +Ve | +Ve | +Ve | +Ve | +Ve | +Ve |
|  | Baggari | Chicken | +Ve | __Ve | +Ve | +Ve | +Ve | +Ve | +Ve | +Ve | +Ve |
|  | Baggari | Chicken | +Ve | __Ve | +Ve | +Ve | +Ve | +Ve | +Ve | +Ve | +Ve |
|  | Baggari | Chicken | +Ve | +Ve | +Ve | +Ve | +Ve | +Ve | +Ve | +Ve | +Ve |
|  | Baggari | Chicken | +Ve | +Ve | +Ve | +Ve | +Ve | +Ve | +Ve | +Ve | +Ve |
|  | Baggari | Humans | +Ve | __Ve | +Ve | +Ve | +Ve | +Ve | +Ve | +Ve | +Ve |
|  | Baggari | Chicken | +Ve | +Ve | +Ve | +Ve | +Ve | +Ve | +Ve | +Ve | +Ve |
|  | Baggari | Chicken | +Ve | +Ve | +Ve | +Ve | +Ve | +Ve | +Ve | +Ve | +Ve |
|  | Busuri | Chicken | +Ve | __Ve | +Ve | +Ve | +Ve | +Ve | +Ve | +Ve | +Ve |
|  | Busuri | Humans | +Ve | +Ve | +Ve | +Ve | +Ve | +Ve | +Ve | +Ve | +Ve |

**Supplementary Materials 3: PCR method targeting the *Salmonella InvA* gene**

| **S/N** | **Sample ID** | **Source** | **Place** | **Colony Morphology** | **PCR *invA* gene** |
| --- | --- | --- | --- | --- | --- |
|  | 14 | Baggari | Human | + | + |
|  | 27 | Baggari | Human | + | + |
|  | 32 | Baggari | Human | + | + |
|  | 37 | Baggari | Human | + | + |
|  | 48 | Baggari | Human | + | + |
|  | 61 | Baggari | Human | + | + |
|  | 66 | Baggari | Human | + | + |
|  | 70 | Baggari | Human | + | + |
|  | 81 | Busuri | Human | + | + |
|  | 95 | Bansuri | Human | + | + |
|  | 96 | Busuri | Human | + | + |
|  | 149 | Baggari | Chicken | + | + |
|  | 157 | Baggari | Chicken | + | + |
|  | 167 | Baggari | Chicken | + | + |
|  | 179 | Baggari | Chicken | + | + |
|  | 186 | Baggari | Chicken | + | + |
|  | 357 | Baggari | Chicken | + | + |
|  | 358 | Baggari | Chicken | + | + |
|  | 380 | Busuri | Chicken | + | + |
|  | 390 | Busuri | Chicken | + | + |

**Supplementary Materials 4**: **Antimicrobial Susceptibility Test results**

| **S/N** | AMP | RES | NA | RES | S | RES | CIP | RES | SXT | RES | C | RES | CN | RES | TE | RES |
| --- | --- | --- | --- | --- | --- | --- | --- | --- | --- | --- | --- | --- | --- | --- | --- | --- |
|  | 17 | S | 22 | S | 20 | S | 30 | S | 25 | S | 22 | S | 19 | S | 17 | M |
|  | 17 | S | 28 | S | 20 | S | 28 | S | 22 | S | 23 | S | 18 | S | 24 | S |
|  | 15 | M | 18 | S | 21 | S | 13 | R | 23 | S | 20 | S | 17 | S | 23 | S |
|  | 03 | R | 20 | S | 23 | S | 30 | S | 25 | S | 20 | S | 19 | S | 17 | S |
|  | 17 | S | 23 | S | 25 | S | 33 | S | 25 | S | 24 | S | 20 | S | 20 | S |
|  | 30 | S | 23 | S | 20 | S | 30 | S | 45 | S | 20 | S | 24 | S | 30 | S |
|  | 21 | S | 20 | S | 25 | S | 29 | S | 20 | S | 20 | S | 18 | S | 11 | R |
|  | 19 | s | 24 | S | 20 | S | 30 | S | 30 | S | 20 | s | 20 | s | 29 | S |
|  | 18 | S | 21 | S | 15 | S | 28 | S | 15 | S | 22 | S | 18 | S | 21 | S |
|  | 22 | S | 20 | S | 22 | S | 30 | S | 22 | S | 20 | S | 20 | S | 22 | S |
|  | 20 | S | 22 | S | 24 | S | 32 | S | 23 | S | 23 | S | 19 | S | 20 | S |
|  | 08 | R | 18 | S | 21 | S | 23 | S | 25 | S | 20 | S | 15 | S | 11 | R |
|  | 20 | S | 25 | S | 25 | S | 30 | S | 24 | S | 23 | S | 20 | S | 20 | S |
|  | 08 | R | 18 | S | 21 | S | 23 | S | 25 | S | 20 | S | 15 | S | 19 | S |
|  | 19 | S | 18 | S | 21 | S | 27 | S | 23 | S | 20 | S | 17 | S | 19 | S |
|  | 20 | S | 20 | S | 21 | S | 23 | S | 27 | S | 22 | S | 16 | S | 13 | R |
|  | 18 | S | 20 | S | 23 | S | 30 | S | 25 | S | 20 | S | 19 | S | 22 | S |
|  | 19 | S | 17 | S | 24 | S | 28 | S | 23 | S | 24 | S | 20 | S | 19 | S |
|  | 20 | S | 20 | S | 23 | S | 30 | S | 25 | S | 23 | S | 20 | S | 20 | S |
|  | 22 | S | 23 | S | 20 | S | 27 | S | 29 | S | 25 | S | 17 | S | 26 | S |

AMP = AMPICILLIN, NA =NALIDIXIC ACID, S =S TRPTOMYCIN, CIP= CIPROFLOXACIN, SXT= TRIMETHOPRIM-SULFAMETHOXAOLE, C= CHLORAMPHENICOL, CN = GENTAMICIN, TE = TETRACYCLINE.

R =RESISTANT; M =INTERMEDIATE and S =SUSCEPTIBLE
